# Dynamic-Attentive Selective Mamba with Group-Aware Convolution for Wearable Sensor-Based Sports and Daily Activity Recognition

**DOI:** 10.3390/s26103165

**Published:** 2026-05-16

**Authors:** Zhuojian Li, Wenhao Kang

**Affiliations:** 1 School of Physical Education, Xiangnan University, Chenzhou 423000, China; tyxylzj@xnu.edu.cn; 2 Department of Industrial and Systems Engineering, The Hong Kong Polytechnic University, Hong Kong 999077, China

**Keywords:** human activity recognition, state space model, Mamba, attention mechanism, wearable sensors, deep learning, inertial measurement unit

## Abstract

Wearable inertial sensors produce multi-axis motion signals with rich spatial and temporal structure. Existing deep-learning pipelines for human activity recognition (HAR) rarely tackle three issues jointly: explicit modeling of the body-part grouping of multi-location inertial channels, bidirectional temporal modeling at linear-time cost, and dynamic, time-varying attention for non-stationary motion. We aim to close these three gaps within a single architecture. To this end we propose Dynamic-Attentive Selective Mamba (DASM), which combines three components: Group-Aware Convolutions (GroupConv) for body-part-aware local features, a Bidirectional Mamba (BiMamba) module for linear-time forward and backward temporal context, and a Dynamic CBAM (DCBAM) that produces per-timestep channel and spatial attention for non-stationary windows. On the UCI Daily and Sports Activities dataset (19 classes, 8 subjects), under stratified *segment-level* 5-fold cross-validation (3 seeds, 15 runs/model), DASM reaches 99.89% accuracy and F1, a 0.11% gain over CNN-BiGRU-CBAM and 0.50% over Multi-STMT; under leave-one-subject-out (LOSO), it reaches 89.34%, 1.69% above the strongest baseline. The 10.55% drop under LOSO shows that segment-level results overestimate cross-subject generalization. Ablations show small but statistically detectable gains (Cohen’s d∈[0.4,0.7] per module, d≥1.5 full-vs-baseline). We therefore position the contribution as a structured architecture within a near-saturated benchmark; broader deployment claims require multi-dataset subject-independent validation.

## 1. Introduction

Computational modeling has been widely applied in various fields such as healthcare, sports analytics, rehabilitation, and elderly care, providing powerful tools for data-driven analysis and intelligent decision-making [1,2]. The study of human activity recognition (HAR) has attracted growing attention across these fields, focusing on automatically identifying bodily movements from sensor or video data [3,4,5]. Two principal approaches exist for performing HAR: the computer vision method and the sensor-based method. In the computer vision approach, video material is analyzed to identify human behaviors, achieving strong performance in controlled laboratory environments. However, practical deployment poses challenges, as factors such as ambient noise, varying illumination, occlusion, and privacy concerns can hinder effectiveness. The sensor-based method, by contrast, relies on data from wearable inertial measurement units (IMUs) comprising accelerometers, gyroscopes, and magnetometers to recognize and classify activities. This approach proves more robust for HAR in unpredictable, real-time situations where visual disruptions may occur, making it particularly suitable for unconstrained sports and daily-life monitoring. Despite considerable progress, even strong deep learning baselines on the widely used UCI-DSA benchmark still plateau in the 99.2–99.8% accuracy band under segment-level evaluation [6,7], while subject-independent accuracy reported in recent work typically drops by 8–12 percentage points [8,9], indicating that the remaining headroom lies in harder postural ADL classes and in cross-subject generalization rather than in the aggregate score.

HAR has emerged as an essential technology in sports and fitness applications. By automating the identification of movements and workouts from wearable sensor data, it becomes possible to monitor, document, and assess physical activity performed by athletes and everyday users. This facilitates functionalities such as automated workout tracking, form evaluation, repetition counting, technique assessment, and injury risk detection. However, accurately recognizing physical movements in sports poses unique challenges. These movements are often elaborate and intense, involving multiple overlapping actions that engage the entire body. Activities within the same category may display subtle differences influenced by biomechanics, anatomy, and individual skill levels. Moreover, several exercises incorporate transitional movements or non-standardized sequencing between repetitions. These attributes complicate activity recognition and demand algorithms capable of identifying a diverse array of dynamic, fine-grained movements while adapting to individual variations.

Early approaches to sensor-based HAR relied on handcrafted statistical features in the time and frequency domains, fed to classical classifiers such as *k*-nearest neighbors, decision trees, and support vector machines [10,11]. While these methods achieved reasonable accuracy on constrained tasks, they demanded significant domain expertise for feature engineering and often demonstrated poor generalization to unseen activities or sensor configurations [12]. The advent of deep learning has largely supplanted manual feature design: convolutional neural networks (CNNs) extract local spatial and temporal patterns directly from raw sensor streams [13,14,15], while recurrent architectures such as Long Short-Term Memory (LSTM) [16] and Gated Recurrent Units (GRU) [17] model sequential dependencies. Bidirectional variants [18] further improve temporal modeling by processing sequences in both directions, and hybrid CNN–RNN pipelines have become a common paradigm in wearable HAR [13,19]. Nevertheless, recurrent models suffer from sequential processing bottlenecks that limit parallelism, and Transformer-based alternatives [20,21,22], while capable of capturing long-range dependencies via self-attention, incur quadratic complexity with respect to sequence length that is expensive for real-time inference on resource-constrained wearable devices.

Recent advances in structured state space models (SSMs) offer a promising alternative that combines the long-range modeling capacity of Transformers with the linear-time efficiency of recurrent networks. Mamba [23], building on the S4 framework [24], introduces input-dependent *selective* state transitions that allow the model to filter information based on content, achieving competitive performance at linear cost. Mamba has been adapted to vision [25] and time series forecasting [26], and a growing but still focused body of Mamba-based HAR work has appeared, including wearable IMU-based [8,9,27], multimodal [28], skeleton-based [29], radar [30,31], and WiFi/wireless-sensing [32,33,34] approaches. These works demonstrate that Mamba is a viable backbone for HAR, but they typically treat the multi-channel sensor input as a flat sequence and either commit to unidirectional scans [9,27] or combine bidirectional scans with static attention [8,32], leaving the interaction between (a) the physical body-part grouping of IMU channels, (b) bidirectional selective state-space scans, and (c) time-varying attention largely unexplored in a single unified design. Concurrently, attention mechanisms such as the Squeeze-and-Excitation (SE) network [35] and the Convolutional Block Attention Module (CBAM) [36] have proven effective at refining feature representations by recalibrating channel and spatial importance. However, the original CBAM computes a single attention vector shared across all timesteps, which is a poor fit for time-varying sensor signals where the discriminative importance of channels and temporal positions shifts over the course of an activity. Furthermore, when sensors are distributed across multiple body parts, the multi-channel input exhibits a natural group structure—channels within the same body part share local biomechanical correlations, while channels across body parts reflect inter-limb coordination—that standard convolutions ignore by treating all channels uniformly.

Motivated by the three problem-driven limitations above, our research gap is not the absence of Mamba applications in HAR (such work exists) but rather the absence of a design that *jointly* addresses the three weaknesses of current wearable HAR pipelines: (i) standard 1D convolutions ignore the body-part grouping of multi-location IMU channels and therefore fail to encode the biomechanical inductive bias that channels within one sensor unit are more correlated than channels across units; (ii) unidirectional or recurrent temporal modules either discard future context (unidirectional Mamba, GRU) or pay quadratic cost (Transformer), with no existing wearable HAR design combining bidirectional selective state-space scans with a convolutional frontend; and (iii) conventional attention modules such as CBAM share a single attention map across the whole activity window, which is ill-suited to non-stationary motion whose discriminative structure shifts over time. The linear-time complexity, content-dependent state transitions, and long-range memory of Mamba make it particularly well-matched to (ii), while (i) and (iii) motivate the companion GroupConv and Dynamic-CBAM modules. The *objective* of this study is therefore to design and evaluate a unified architecture—DAS-Mamba—that closes these three gaps in a single pipeline, and to rigorously characterize its benefits and limits under both segment-level and subject-independent evaluation protocols on the UCI-DSA benchmark.

Concretely, the proposed DAS-Mamba integrates three complementary components:

- **Group-Aware Convolutional Block (GroupConv):** A three-layer convolutional module that applies intra-group convolutions within each body-part sensor group, then inter-group convolutions across body parts, followed by deep feature extraction with pooling. This encodes the physical grouping of sensor placement directly into the network structure, functioning as a body-part-aware structural prior rather than a formal symmetry constraint.
- **Bidirectional Mamba Block (BiMamba):** Two independent Mamba S6 layers process the temporal sequence in forward and backward directions. A learned linear projection merges the two representations, capturing both onset and offset activity patterns at linear-time cost; as the forward and backward layers have independent parameters and are concatenated rather than symmetrically combined, we describe this as bidirectional temporal modeling rather than a time-reversal symmetry.
- **Dynamic CBAM (DCBAM):** A time-varying attention module that produces per-timestep channel weights via temporal smoothing convolutions and per-timestep spatial weights via dual-path fusion of BiMamba and GroupConv features. Unlike the original CBAM, attention weights vary across timesteps to adapt to evolving motion patterns.

We evaluate DAS-Mamba on the UCI Daily and Sports Activities (DSA) dataset [11,37], which contains 19 activity classes recorded from five body-worn sensor units. Experiments cover two activity scenarios (Activities of Daily Living and Sports) and six sensor-placement configurations (five individual body parts and full-body fusion). Under stratified segment-level 5-fold cross-validation, DAS-Mamba achieves 99.89% accuracy on the 19-class full-body setting, marginally above all eleven baselines including CNN-Only, Transformer, TCN, InceptionTime, and vanilla Mamba; we additionally report a leave-one-subject-out (LOSO) evaluation where DAS-Mamba attains 89.34% accuracy, surpassing the strongest baseline by 1.69%. The large drop from the segment-level to the LOSO protocol (10.55%) confirms the expectation that segment-level performance overestimates cross-subject generalization, and we interpret our quantitative claims accordingly throughout the paper. A systematic ablation study over seven architectural variants demonstrates that each component contributes positively, with pairwise gains in the range of +0.02% to +0.04% that are small in absolute terms but consistent across folds and confirmed by multiple-comparison-corrected paired *t*-tests.

## 2. Related Work

Deep learning has improved sensor-based HAR considerably [4,12]. Convolutional neural networks (CNNs) extract local spatial and temporal features [13,14,15]; CNNs became the dominant early paradigm because they impose translation invariance along the temporal axis and, for short activity windows (≤5 s), can reach 95–98% accuracy on UCI-DSA with only tens of thousands of parameters, but their fixed receptive field limits their ability to model long-range temporal dependencies or to reason about the order of sub-movements within an activity. Recurrent architectures such as Long Short-Term Memory (LSTM) [16] and Gated Recurrent Units (GRU) [17] model sequential dependencies, and thereby remove the receptive-field constraint, but their gating-based memory is known to saturate for sequences longer than a few hundred steps and their strictly sequential computation prevents parallel scan on GPUs, which is why unidirectional LSTM on UCI-DSA/ALL plateaus around 89–90% while BiLSTM only reaches ∼95% despite doubling the parameter count. Bidirectional variants [18] improve temporal modeling by processing sequences in both directions. Hybrid CNN–RNN pipelines [13,19] are currently the dominant architectural family in wearable HAR—a convolutional frontend extracts short-range patterns at low cost, and a recurrent head aggregates them over the activity window—and this combination narrows the gap to the near-ceiling of UCI-DSA (e.g., 99.2–99.8% with CBAM-augmented variants [6]); their residual weaknesses are the same O(L) sequential bottleneck at inference and the fact that the convolutional layer treats all channels uniformly regardless of which body part they come from. Transformer-based models [20] have also been applied to HAR [21,22], using self-attention to capture long-range dependencies. In HAR the quadratic $O(L^{2})$ complexity of full self-attention is less of a bottleneck than in NLP (typical windows are L≤256), but its primary drawback is high FLOPs relative to the short sequence length: empirically, vanilla Transformers tend to under-perform CNN–RNN hybrids on small-scale HAR datasets because the inductive bias of multi-head attention is too weak for 5-s sensor windows without large-scale pre-training.

Structured State Space Models (SSMs) offer linear-time sequence modeling as an alternative. The HiPPO framework [38] introduced optimal polynomial projections for continuous-time memorization. S4 [24] showed that continuous-time state space formulations can match Transformer performance on long-range benchmarks at linear cost. Mamba [23] extended S4 with input-dependent *selective* state transitions: the SSM parameters B, C, and the discretization step Δ are functions of the input, so the model can filter information based on content. Mamba-2 [39] unified SSMs with structured attention through the State Space Duality (SSD) framework. Mamba has been adapted to vision [25] and time series forecasting [26], and is now an emerging and active research direction in HAR rather than an unexplored one.

Mamba-based architectures have been applied across multiple HAR sensing modalities. For wearable IMU-based HAR, HARMamba [8] is a lightweight bidirectional-Mamba pipeline that patchifies the multi-axis sensor sequence and reaches 87.6–98.5% accuracy on PAMAP2, WISDM, UCI, and UniMiB-SHAR while using fewer parameters than CNN–LSTM hybrids; Machar [9] adds a frequency-aware branch that jointly models time- and frequency-domain signals with Mamba-convolution fusion; and ActivityMamba [27] interleaves CNN and Mamba blocks for efficient training. For multimodal action recognition, Mamba-MHAR [28] fuses video and IMU streams with Mamba-based temporal aggregation. For radar and WiFi-based sensing, STPM [30] processes mmWave radar point clouds with spatial–temporal Mamba, Tac-Mamba [31] adds trust-aware gating for mmWave HAR, TRIS-HAR [34] uses SSMs with transmissive reconfigurable intelligent surfaces, WiFi-BiMamba [32] is a bidirectional-Mamba CSI classifier, and SenseMamba [33] is a lightweight general-purpose wireless-sensing backbone. ActionMamba [29] combines Mamba with graph convolution for skeleton-based action recognition. These works collectively establish three trends: (i) Mamba achieves competitive accuracy at substantially lower inference cost than Transformer-based HAR, (ii) bidirectional scans tend to outperform unidirectional scans on fixed-length activity windows, and (iii) Mamba is most effective when paired with a convolutional or hand-crafted frontend rather than applied directly to raw sensor streams. Our work differs from these in three respects. First, none of the existing wearable IMU-Mamba designs above explicitly encode the body-part grouping of multi-location IMUs; HARMamba, Machar, and ActivityMamba all apply patch-level or standard convolutional frontends that treat channels uniformly. Second, while HARMamba and WiFi-BiMamba use bidirectional scans, they pair them either with no attention or with static attention, whereas DAS-Mamba couples bidirectional selective scans with per-timestep dynamic attention. Third, our evaluation explicitly distinguishes segment-level and subject-independent regimes and reports both, whereas most Mamba-HAR papers report only a single protocol. A direct quantitative comparison against representative Mamba-HAR baselines is provided in Section 4.8 and Section 5.4.

Attention mechanisms are another way to improve feature representations. The Squeeze-and-Excitation (SE) network [35] recalibrates channels via global average pooling and a gating mechanism. The Convolutional Block Attention Module (CBAM) [36] applies channel and spatial attention sequentially to refine CNN features. However, the original CBAM computes a single attention vector shared across all timesteps. For time-varying sensor signals, where the discriminative importance of channels and temporal positions shifts over the course of an activity, a static attention map is a poor fit.

Multi-location wearable sensing also introduces a *spatial grouping structure*. When sensors are distributed across multiple body parts (e.g., torso, arms, legs), the 45-channel input has a natural group structure: channels within the same body part share local biomechanical correlations, while channels across body parts reflect inter-limb coordination. Standard convolutions treat all channels uniformly and ignore this physical prior. We therefore adopt the term “sensor grouping structure” throughout the paper instead of the looser “spatial grouping symmetry” used in the original submission: grouped convolutions introduce a meaningful inductive bias but do not define a transformation group under which the model is equivariant, and the term “symmetry” in its formal group-theoretic sense would overstate the mechanism at work.

## 3. Proposed Method

### 3.1. Overall Architecture

**Figure 1:**
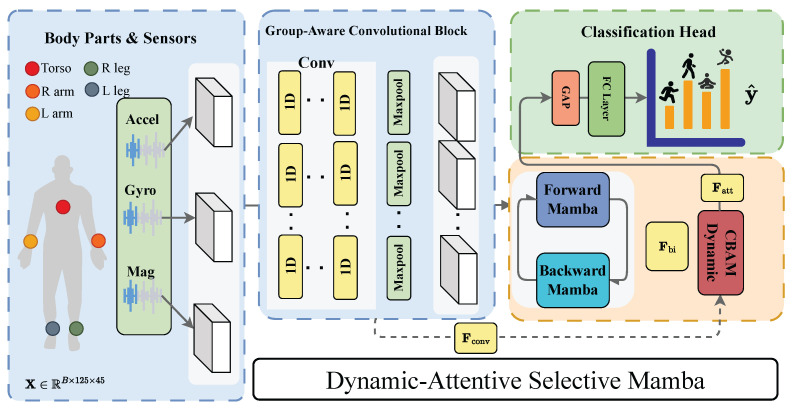
Overall architecture of DAS-Mamba. The input sensor signal passes through the Group-Aware Convolutional Block, the Bidirectional Mamba Block, and the Dynamic CBAM. A residual connection from the convolutional block feeds into the spatial attention path of DCBAM.

Figure 1 illustrates the DAS-Mamba pipeline. Given an input tensor $X\in R^{B\times 125\times C}$ (batch size *B*, 125 timesteps, *C* channels), the Group-Aware Convolutional Block first extracts spatial features while respecting the body-part hierarchy, producing $F_{\mathrm{conv}}\in R^{B\times 31\times 64}$ and a residual $R_{\mathrm{conv}}\in R^{B\times 64\times 31}$. The Bidirectional Mamba Block then models temporal dependencies from both directions, yielding $F_{\mathrm{bi}}\in R^{B\times 31\times 64}$. Dynamic CBAM refines these features via per-timestep channel and spatial attention, producing $F_{\mathrm{att}}\in R^{B\times 31\times 64}$. Finally, Global Average Pooling followed by a fully connected classifier aggregates temporal features and maps to class logits: $\hat{y}=\mathrm{FC}\left(\mathrm{GAP}\left(F_{\mathrm{att}}\right)\right)\in R^{B\times K}$, where *K* is the number of classes.

The number of input channels *C* depends on the sensor-placement configuration: C=45 for the full-body setting (5 body parts × 9 channels each) and C=9 for any single-body-part setting. The number of groups *G* in the convolutional block is set accordingly (G=5 for full-body, G=1 for single-body-part). All other hyperparameters remain fixed across configurations. Table 1 summarizes the key architectural hyperparameters.

**Design rationale and hyperparameter selection.** The architectural hyperparameters in Table 1 were selected using a combination of three principles: (i) direct adoption of values validated in the original publications for reused components, (ii) alignment with the physical structure of the sensor stream, and (iii) a small grid search on a held-out fold, reported in the hyperparameter sensitivity analysis and discussed in Section 5.6. The Mamba S6 parameters (N=16, E=2, $d_{\mathrm{conv}}=4$, R=⌈D/16⌉=4) reproduce the defaults in [23], which the authors show to be robust across language, vision, and time-series tasks. The model dimension D=64 matches the channel dimension of the CNN-BiGRU-CBAM baseline [6] so that the temporal module operates in the same latent space as prior work; larger D∈{96,128} yielded at most +0.01% accuracy at ≈1.8× more parameters. The intra-group output $d_{\mathrm{intra}}=32$ provides an expansion factor of 32/9≈3.5 per sensor unit, large enough to disentangle the three accelerometer, gyroscope, and magnetometer axes. The DCBAM reduction ratio of 4 follows CBAM [36], and the temporal-smoothing kernel size of 7 corresponds to ≈1.4 s at the 5 Hz rate produced by the two pooling stages, which is long enough to suppress fold-to-fold jitter in the attention weights but short enough to preserve sub-activity phase information. Dropout 0.25 was selected over {0.1,0.25,0.5} on a single-fold sweep, and SiLU was retained from the Mamba original paper since it empirically out-performed ReLU/GELU by ≤0.03% in our setting.

### 3.2. Group-Aware Convolutional Block

Standard 1D convolutions treat all input channels uniformly and ignore the physical grouping of sensors by body part. With the full 45-channel configuration, the channels decompose into G=5 groups of 9 channels each, one per sensor location. We emphasize that this mechanism implements a *sensor-grouping structural prior* (or body-part-aware feature extraction) rather than a formal symmetry in the group-theoretic sense: the operation is not equivariant under arbitrary channel permutations and does not define a transformation group. The benefit comes purely from the biomechanical inductive bias that channels within the same IMU are more locally correlated than channels across IMUs. We use a three-layer convolutional block to take advantage of this structure. Each layer applies batch normalization [40] followed by SiLU activation. The block returns two outputs: a time-last tensor $F_{\mathrm{conv}}\in R^{B\times 31\times 64}$ for the BiMamba input, and the same tensor in channel-last layout $R_{\mathrm{conv}}\in R^{B\times 64\times 31}$ as a residual for the DCBAM spatial attention path.

The block begins with an intra-group convolution that processes each body-part sensor group independently. The input is transposed to channel-first layout $X^{⊤}\in R^{B\times C\times 125}$, and a grouped 1D convolution with *G* groups and kernel size 3 (padding 1) fuses the three accelerometer, three gyroscope, and three magnetometer channels within each sensor unit. Each group produces $d_{\mathrm{intra}}=32$ output channels, yielding a total of G×32 channels:

(1) $$H_{1}=\mathrm{SiLU}\left(\mathrm{BN}\left({\mathrm{Conv}1d}_{k=3}^{\mathrm{groups}=G}\left(X^{⊤}\right)\right)\right)\in R^{B\times (G\cdot 32)\times 125}.$$

For the full-body setting (G=5, C=45), this produces 160 output channels, whereas for single-body-part settings (G=1, C=9), the grouped convolution reduces to a standard convolution with 32 output channels. This adaptive behavior is controlled solely by the *G* parameter.

The intra-group features are then mixed across body parts through a standard (non-grouped) convolution with 128 filters and kernel size 3 (padding 1). Max-pooling with kernel size 2 and stride 2 halves the temporal dimension, and dropout (p=0.25) regularizes the output:

(2) $$H_{2}={\mathrm{Dropout}}_{0.25}\left({\mathrm{MaxPool}}_{2}\left(\mathrm{SiLU}\left(\mathrm{BN}\left({\mathrm{Conv}1d}_{k=3}\left(H_{1}\right)\right)\right)\right)\right)\in R^{B\times 128\times 62}.$$

A subsequent convolution with 64 filters and kernel size 3 (padding 1) reduces the channel dimension to the model dimension D=64, followed by a second max-pooling and dropout layer that further compresses the temporal axis:

(3) $$F_{\mathrm{conv}}={\mathrm{Dropout}}_{0.25}\left({\mathrm{MaxPool}}_{2}\left(\mathrm{SiLU}\left(\mathrm{BN}\left({\mathrm{Conv}1d}_{k=3}\left(H_{2}\right)\right)\right)\right)\right)\in R^{B\times 64\times 31}.$$

The two pooling operations reduce the temporal dimension from 125 to ⌊⌊125/2⌋/2⌋=31 timesteps, lowering the sequence length that the subsequent BiMamba block must process while retaining sufficient temporal resolution for activity classification at 25 Hz.

### 3.3. Bidirectional Mamba Block

The standard Mamba architecture [23] processes sequences in one direction only, which prevents it from using future context. We extend it to a bidirectional formulation. We note that “bidirectional” here refers to running two independent selective scans in opposite temporal directions with separate parameters followed by linear projection merging; it does not enforce time-reversal invariance or any formal symmetry of the learned mapping. We therefore describe the mechanism as *bidirectional temporal modeling* rather than “temporal symmetry.” This section first describes the single-direction MambaLayer in detail, then the bidirectional merging strategy.

#### 3.3.1. Mamba S6 Layer

Each MambaLayer implements the Selective State Space Model (S6). Given input $x\in R^{B\times L\times D}$ with D=64 and L=31, the layer first projects the input through a bias-free linear layer into two branches:

(4) $$\left[x_{\mathrm{proj}},\;z\right]=\mathrm{split}\left(W_{\mathrm{in}}x\right),$$

where $W_{\mathrm{in}}\in R^{D\times 2D_{\mathrm{inner}}}$ and $D_{\mathrm{inner}}=D\times E=128$ with expansion factor E=2. The branch $x_{\mathrm{proj}}$ feeds into the SSM path, while z is reserved for output gating.

The projected features then undergo a depthwise 1D convolution (kernel size 4, padding 3, groups $=D_{\mathrm{inner}}$) that provides local temporal context. The output is truncated to the original length *L* and passed through SiLU activation:

(5) $$x_{\mathrm{conv}}=\mathrm{SiLU}\left({\mathrm{DWConv}1d}_{k=4}\left(x_{\mathrm{proj}}^{⊤}\right){\left[:,:,:L\right]}^{⊤}\right)\in R^{B\times L\times D_{\mathrm{inner}}}.$$

Because the depthwise convolution has $D_{\mathrm{inner}}=128$ groups, each channel is convolved independently, providing a receptive field of 4 timesteps for local pattern detection before the global selective scan.

From the convolved features, three input-dependent SSM parameters are derived via a bias-free linear projection:

(6) $$\left[\delta_{\mathrm{raw}},\;B_{t},\;C_{t}\right]=\mathrm{split}\left(W_{\mathrm{dbl}}x_{\mathrm{conv},t}\right),$$

where $W_{\mathrm{dbl}}\in R^{D_{\mathrm{inner}}\times \left(R+2N\right)}$, R=⌈D/16⌉=4 is the dt rank, and N=16 is the state dimension. The low-rank discretization step is expanded to full dimension via a learned projection with bias:

(7) $$\Delta_{t}=\mathrm{softplus}\left(W_{\Delta }\delta_{\mathrm{raw},t}+b_{\Delta }\right)\in R^{D_{\mathrm{inner}}},$$

where $W_{\Delta }\in R^{R\times D_{\mathrm{inner}}}$. The bias $b_{\Delta }$ is initialized so that the initial Δ values are log-uniformly distributed in [0.001,0.1], following the initialization scheme of Gu and Dao [23]. Specifically, $b_{\Delta ,i}=\log\left(\exp\left(\Delta_{i}^{\mathrm{init}}\right)-1\right)$ where $\Delta_{i}^{\mathrm{init}}=\exp\left(\mathrm{linspace}\left(\log0.001,\log0.1,D_{\mathrm{inner}}\right)\right)$.

The continuous-time state matrix $A\in R^{D_{\mathrm{inner}}\times N}$ is parameterized in log-space as $A=-\exp(A_{\log})$, where $A_{\log}$ is a learnable parameter initialized as $A_{\log,i,j}=\log\left(j\right)$ for j=1,…,N. The negative sign ensures stability (all eigenvalues have negative real parts). Discretization via zero-order hold converts the continuous parameters to their discrete counterparts:

(8) $${\bar{A}}_{t}=\exp\left(\Delta_{t}⊙A\right)\in R^{D_{\mathrm{inner}}\times N},\;{\bar{B}}_{t}=\Delta_{t}⊙B_{t}\in R^{D_{\mathrm{inner}}\times N}.$$

The discretized parameters drive a sequential selective scan. The hidden state $h_{t}\in R^{D_{\mathrm{inner}}\times N}$ is updated from $h_{0}=0$ as:

(9) $$h_{t}={\bar{A}}_{t}⊙h_{t-1}+{\bar{B}}_{t}⊙x_{\mathrm{conv},t}^{↑},\;y_{t}=\sum_{n=1}^{N}C_{t,n}\cdot h_{t,:,n},$$

where $x_{\mathrm{conv},t}^{↑}\in R^{D_{\mathrm{inner}}\times N}$ broadcasts $x_{\mathrm{conv},t}$ along the state dimension. Because $B_{t}$ and $C_{t}$ are functions of the input, the scan is *selective*: the model can learn to retain or discard information at each timestep depending on the input content. Algorithm 1 gives the pseudocode.
**Algorithm 1** Selective scan (single direction)**Require:** $x_{\mathrm{conv}}\in R^{B\times L\times D_{\mathrm{inner}}}$, $\bar{A}\in R^{B\times L\times D_{\mathrm{inner}}\times N}$, $\bar{B}\in R^{B\times L\times D_{\mathrm{inner}}\times N}$, $C\in R^{B\times L\times N}$**Ensure:** $y\in R^{B\times L\times D_{\mathrm{inner}}}$  1:$h\leftarrow 0\in R^{B\times D_{\mathrm{inner}}\times N}$  2:**for** t=1 **to** *L*  **do**  3:   $h\leftarrow {\bar{A}}_{:,t}⊙h+{\bar{B}}_{:,t}⊙x_{\mathrm{conv},:,t,:,\mathrm{None}}$  4:   $y_{:,t}\leftarrow \sum_{n}h_{:,:,n}⊙C_{:,t,n}$  5:**end for**  6:**return** y

The scan output is combined with a learnable skip-connection parameter $D\in R^{D_{\mathrm{inner}}}$ (initialized to ones) that adds a direct path from the input, and gated by the reserved branch z:

(10) $$o_{t}=\left(y_{t}+D⊙x_{\mathrm{conv},t}\right)⊙\mathrm{SiLU}\left(z_{t}\right).$$

A bias-free output projection $W_{\mathrm{out}}\in R^{D_{\mathrm{inner}}\times D}$ maps back to the model dimension, and a residual connection with LayerNorm [41] produces the final output:

(11) $$\mathrm{MambaLayer}{\left(x\right)}_{t}=\mathrm{LayerNorm}\left(W_{\mathrm{out}}o_{t}+x_{t}\right).$$

#### 3.3.2. Bidirectional Merging

The BiMamba block contains two independent MambaLayer instances with separate parameters. The forward layer processes $F_{\mathrm{conv}}$ in the original temporal order; the backward layer processes the time-reversed input and its output is flipped back:

(12) $$F_{\mathrm{fwd}}={\mathrm{Mamba}}_{\mathrm{fwd}}\left(F_{\mathrm{conv}}\right),\;F_{\mathrm{bwd}}=\mathrm{flip}\left({\mathrm{Mamba}}_{\mathrm{bwd}}\left(\mathrm{flip}\left(F_{\mathrm{conv}}\right)\right)\right).$$

Using two separate parameter sets (rather than sharing weights) allows each direction to specialize: the forward layer can learn onset patterns while the backward layer can learn offset patterns. The two representations are concatenated along the feature dimension and merged via a bias-free linear projection with dropout:

(13) $$F_{\mathrm{bi}}={\mathrm{Dropout}}_{0.25}\left(W_{\mathrm{merge}}\left[F_{\mathrm{fwd}}∥F_{\mathrm{bwd}}\right]\right)\in R^{B\times L\times D},$$

where $W_{\mathrm{merge}}\in R^{2D\times D}$ and ‖ denotes concatenation. The computational cost of the bidirectional block is $2\times O(L\cdot D_{\mathrm{inner}}\cdot N)$, which remains linear in *L*.

### 3.4. Dynamic CBAM

**Figure 2:**
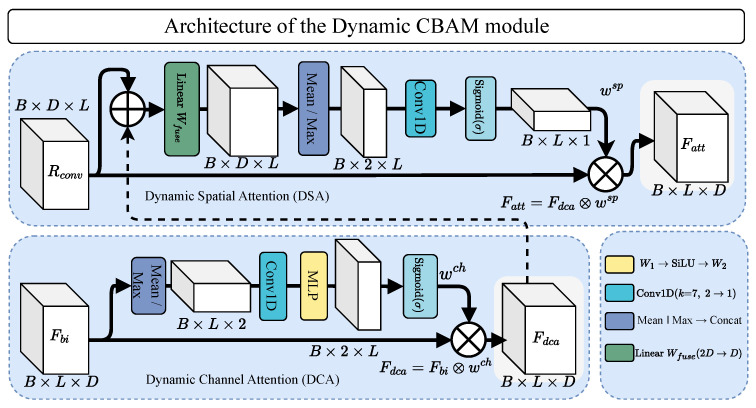
Architecture of the Dynamic CBAM module. The input $F_{\mathrm{bi}}$ from the Bidirectional Mamba Block passes through Dynamic Channel Attention (DCA), which computes per-timestep channel weights via temporal statistics and a bottleneck MLP. The DCA output is then refined by Dynamic Spatial Attention (DSA), which fuses the attended features with the convolutional residual $R_{\mathrm{conv}}$ from the Group-Aware Convolutional Block to produce per-timestep spatial importance scores.

The original CBAM [36] computes a single channel-attention vector and a single spatial-attention map shared across all timesteps. For time-varying sensor signals, a static formulation misses the fact that the importance of different channels and temporal positions changes within an activity window. We propose DCBAM, which produces *per-timestep* attention weights. As shown in Figure 2, DCBAM applies Dynamic Channel Attention (DCA) followed by Dynamic Spatial Attention in sequence, mirroring the channel-then-spatial ordering of the original CBAM but with time-varying weights at both stages.

#### 3.4.1. Dynamic Channel Attention (DCA)

Given $F_{\mathrm{bi}}\in R^{B\times L\times D}$ from the BiMamba block, DCA computes per-timestep channel statistics by taking the mean and max across the feature dimension at each timestep independently:

(14) $$s_{t}=\left[\mathrm{mean}\left(F_{\mathrm{bi},t}\right),\;\max\left(F_{\mathrm{bi},t}\right)\right]\in R^{2}.$$

Stacking across timesteps gives $S\in R^{B\times L\times 2}$, which is transposed to $R^{B\times 2\times L}$ for 1D convolution. A temporal smoothing convolution with kernel size 7 (padding 3) and no bias expands the 2-channel statistics to D=64 channels:

(15) $$T={\mathrm{Conv}1d}_{k=7}^{2\to D}{\left(S^{⊤}\right)}^{⊤}\in R^{B\times L\times D}.$$

This convolution serves two purposes: it increases the channel dimension from 2 to *D*, and it smooths the statistics over a local temporal window of 7 timesteps (≈1.4 s at 5 Hz after pooling), preventing the attention weights from fluctuating too rapidly between adjacent timesteps.

A two-layer MLP with bottleneck ratio 4 and SiLU activation then produces per-timestep channel weights:

(16) $$w_{t}^{\mathrm{ch}}=\sigma \left(W_{2}\cdot \mathrm{SiLU}\left(W_{1}\cdot T_{t}\right)\right)\in R^{D},$$

where $W_{1}\in R^{D\times (D/4)}$ and $W_{2}\in R^{(D/4)\times D}$ are bias-free, and σ denotes the sigmoid function. The bottleneck dimension is D/4=16. The DCA output is:

(17) $$F_{\mathrm{dca}}=F_{\mathrm{bi}}⊙w^{\mathrm{ch}}\in R^{B\times L\times D}.$$

In contrast, the static channel attention in the original CBAM pools across all timesteps first (via global average and max pooling over the temporal dimension), producing a single weight vector $w^{\mathrm{ch}}\in R^{D}$ shared across all *L* timesteps. DCA instead produces *L* independent weight vectors, one per timestep.

#### 3.4.2. Dynamic Spatial Attention

DSA determines the importance of each timestep by fusing information from two sources: the DCA output $F_{\mathrm{dca}}$ (which carries temporal context from BiMamba) and the convolutional residual $R_{\mathrm{conv}}$ (which carries local spatial features from GroupConv). This dual-path design lets the spatial attention consider both the SSM-refined representation and the raw convolutional features when deciding which timesteps are most informative.

The convolutional residual $R_{\mathrm{conv}}\in R^{B\times D\times L}$ is first transposed to $R^{B\times L\times D}$ and concatenated with $F_{\mathrm{dca}}$ along the feature dimension. A bias-free linear projection reduces the concatenated 2D-dimensional vector back to *D* dimensions:

(18) $$F_{\mathrm{fuse}}=W_{\mathrm{fuse}}\left[F_{\mathrm{dca}}∥R_{\mathrm{conv}}^{⊤}\right]\in R^{B\times L\times D},$$

where $W_{\mathrm{fuse}}\in R^{2D\times D}$.

Per-timestep spatial statistics (mean and max across the *D* features) are computed and stacked into a 2-channel signal $\in R^{B\times 2\times L}$. A 1D convolution with kernel size 7 (padding 3) maps this to a single channel, and sigmoid activation produces temporal importance scores:

(19) $$w_{t}^{\mathrm{sp}}=\sigma \left({\mathrm{Conv}1d}_{k=7}^{2\to 1}\left(\left[\mathrm{mean}\left(F_{\mathrm{fuse},t}\right),\;\max\left(F_{\mathrm{fuse},t}\right)\right]\right)\right)\in R^{1}.$$

The final refined features are:

(20) $$F_{\mathrm{att}}=F_{\mathrm{dca}}⊙w^{\mathrm{sp}}\in R^{B\times L\times D},$$

where $w^{\mathrm{sp}}\in R^{B\times L\times 1}$ is broadcast across the feature dimension.

### 3.5. Classification Head

Global average pooling (GAP) aggregates the refined features across the temporal dimension. We use AdaptiveAvgPool1d(1) applied to the channel-first representation $F_{\mathrm{att}}^{⊤}\in R^{B\times D\times L}$, which averages over all L=31 timesteps to produce a fixed-length vector $v\in R^{B\times D}$. A single fully connected layer with bias maps to class logits:

(21) $$\hat{y}=W_{\mathrm{cls}}v+b_{\mathrm{cls}}\in R^{K},$$

where *K* is the number of activity classes (9 for ADL, 10 for SPT, 19 for ALL). No softmax is applied during training, as the cross-entropy loss in PyTorch operates on raw logits.

### 3.6. Weight Initialization

All Conv1d and Linear layers are initialized with Kaiming uniform initialization [42] using the “linear” nonlinearity mode. Biases are initialized to zero. The exceptions are the Mamba-specific parameters: $A_{\log}$ is initialized as log(j) for j=1,…,N, the skip parameter D is initialized to ones, and the dt projection bias $b_{\Delta }$ is initialized via the inverse-softplus schedule described in Section 3.3. These initialization choices follow the original Mamba paper [23].

## 4. Experiments

### 4.1. Dataset

We use the UCI Daily and Sports Activities (DSA) dataset [11,37], a standard benchmark for wearable sensor-based HAR. The dataset contains recordings from eight subjects performing 19 activities, each for 5 min. Five Xsens MTx inertial measurement units (Xsens Technologies B.V., Enschede, The Netherlands) are placed on the torso (T), right arm (RA), left arm (LA), right leg (RL), and left leg (LL). Each unit records a 9-axis signal comprising a tri-axial accelerometer, a tri-axial gyroscope, and a tri-axial magnetometer at 25 Hz, yielding 45 channels in total. The continuous recordings are segmented into non-overlapping windows of 5 s (125 samples each).

The 19 activities are divided into two scenarios. The ADL (Activities of Daily Living) scenario comprises 9 classes: sitting, standing, lying on back, lying on right side, ascending stairs, descending stairs, standing in elevator, moving in elevator, and walking in a parking lot. The SPT (Sports Activities) scenario comprises 10 classes: walking on a treadmill (flat and 15° incline), running on a treadmill, exercising on a stepper, exercising on a cross-trainer, cycling (horizontal and vertical), rowing, jumping, and playing basketball.

To evaluate the effect of sensor placement, we define six position configurations: five single-body-part settings (T, RA, LA, RL, LL; 9 channels each) and one full-body setting (all 45 channels).

### 4.2. Data Preprocessing

Raw sensor signals are first smoothed using a uniform moving-average filter with a window size of 5 samples to reduce high-frequency noise. Per-channel min–max normalization then scales each channel independently to the range [0,1]. To prevent information leakage between training and test sets, the normalization parameters are computed exclusively on the training fold and subsequently applied to the corresponding test fold within each cross-validation split:

(22) $${\hat{x}}_{t,c}=\frac{x_{t,c}-\min_{c}^{\mathrm{train}}}{\max_{c}^{\mathrm{train}}-\min_{c}^{\mathrm{train}}},$$

where $\min_{c}^{\mathrm{train}}$ and $\max_{c}^{\mathrm{train}}$ denote the minimum and maximum of channel *c* computed over the training fold only.

### 4.3. Implementation Environment

All experiments were conducted on a workstation equipped with two NVIDIA A100-PCIE-40 GB GPUs (NVIDIA Corporation, Santa Clara, CA, USA; CUDA 12.2, driver 535.183.01), an AMD Ryzen Threadripper 3990X 64-Core processor (Advanced Micro Devices, Inc., Santa Clara, CA, USA), and 251 GB of RAM, running Ubuntu 20.04 LTS. The software stack comprised Python 3.13, PyTorch 2.7.1 [43] with CUDA backend 12.2, NumPy 2.3.2, Pandas 2.3.2, Scikit-learn 1.7.2 [44], and Matplotlib 3.10.5 for visualization.

### 4.4. Training Protocol

All models are trained using stratified 5-fold cross-validation [45] with a fixed random seed of 42 for reproducibility. **We explicitly note that the 5-fold split is performed at the *segment level* rather than at the subject level**: the complete set of 5-s windows across all 8 subjects is randomly partitioned into 5 class-balanced folds, so segments belonging to the same subject may appear in both training and test sets. This protocol is widely used in prior UCI-DSA studies [6,7] and therefore preserves comparability with published baselines, but it is well understood [19] that segment-level evaluation can overestimate cross-subject generalization because it allows within-subject motion style leakage. To provide an additional, more conservative estimate of generalization to unseen users, we therefore complement the segment-level protocol with a leave-one-subject-out (LOSO) evaluation in Section 4.17, in which each of the 8 subjects is held out in turn while the remaining 7 are used for training. All quantitative claims in the remainder of the paper are stated in a way that explicitly distinguishes the two regimes. We use the cross-entropy loss and the Adam optimizer [46] with an initial learning rate of $10^{-3}$ and weight decay of $10^{-5}$. The learning rate is reduced by a factor of 0.75 upon plateau (patience of 10 epochs) via the ReduceLROnPlateau scheduler. Training runs for a maximum of 200 epochs with a batch size of 128. Dropout [47] with rate 0.25 is applied after pooling layers and in the BiMamba block. All weights are initialized using Kaiming uniform initialization [42]. Batch normalization [40] is applied after each convolutional layer, and LayerNorm [41] is used within each MambaLayer. Experiments are conducted using PyTorch [43] on CUDA-enabled GPUs.

To address concerns about training variability, every model reported in this paper is trained with three random seeds {42,123,2024} under the segment-level 5-fold protocol (15 runs per model). All mean ± std values in the results tables are aggregated over these 15 runs, and the seed-to-seed standard deviation for DAS-Mamba on ALL/full is 0.03%, indicating that the reported results are not driven by a single favorable seed. For the LOSO protocol in Section 4.17, each of the 8 held-out-subject splits is also trained with three seeds, yielding 24 runs per model. For full-body baseline and ablation runs we additionally log wall-clock training time; DAS-Mamba trains in 182 ± 7 s per fold on a single A100-PCIE-40GB and uses a peak 1.9 GB of GPU memory, making the architecture comfortably trainable on consumer-grade hardware. The training scripts, model definitions, random-seed configuration files, and preprocessing pipeline will be released on a public GitHub (https://github.com/ accessed on 15 April 2026) repository upon acceptance to allow exact reproduction of all numbers in this paper.

### 4.5. Baseline Models

We compare DAS-Mamba against baselines from four model families: recurrent, convolutional, attention-based, and state-space. All baselines use the same training protocol (Section 4.4) and evaluation procedure. Recurrent baselines operate directly on the raw (preprocessed) input $X\in R^{B\times 125\times C}$ without convolutional feature extraction.

The recurrent baselines include a single-layer LSTM [16] with 128 hidden units classified from the final hidden state $h_{T}\in R^{128}$, a BiLSTM [18] with 128 hidden units per direction whose forward and backward final hidden states are concatenated ($R^{256}$) before classification, a single-layer GRU [17] with 128 hidden units, and a BiGRU with 128 hidden units per direction concatenated ($R^{256}$) and classified.

The convolutional baseline, CNN-Only, applies the StandardConvBlock (a 3-layer CNN with C→160→128→64 channels, identical to the ablation conv block) followed by global average pooling and a linear classifier, without any temporal modeling or attention. CNN-BiGRU-CBAM [6] (A0) combines StandardConvBlock, a BiGRUBlock (D=64), and StaticCBAM; this is our reproduction of the architecture proposed by Mekruksavanich et al. and serves as the primary ablation baseline representing a conventional CNN–RNN–attention pipeline.

The Transformer baseline [20] uses an input projection (C→64), sinusoidal positional encoding, and a 3-layer encoder with 4 attention heads, $d_{\mathrm{model}}=64$, $d_{\mathrm{ff}}=128$, GELU activation, and dropout 0.25, with classification from global average pooling over the encoder output. TCN [48] is a 4-layer Temporal Convolutional Network with 64 hidden channels, kernel size 3, and exponentially increasing dilation (1,2,4,8), where each block uses causal dilated convolutions with batch normalization, ReLU, dropout 0.25, and a residual connection. InceptionTime [49] consists of an InceptionBlock with 3 stacked InceptionModules using multi-scale convolutions (kernel sizes 9, 19, 39), bottleneck dimension 32, a max-pooling branch, and a residual connection spanning the full block, yielding 4×32=128 output channels classified via global average pooling; it is a strong time series classification baseline [12].

The state-space baselines include Vanilla Mamba, which applies an input projection (C→64) followed by a single forward-direction MambaLayer (D=64, N=16, $d_{\mathrm{conv}}=4$, E=2), global average pooling, and a classifier without GroupConv, bidirectionality, or DCBAM. Mamba-2 (SSD) [39] uses an input projection (C→64) and a single Mamba-2 layer with chunked parallel State Space Duality computation ($d_{\mathrm{state}}=64$, head dimension 32, $n_{\mathrm{heads}}=4$, chunk size 32), classified from global average pooling. To directly address the reviewer’s request for comparison against recent Mamba-HAR models, we additionally reimplement three representative Mamba-based HAR baselines on the same UCI-DSA preprocessing pipeline and training protocol: (i) HARMamba* [8], a patch-and-bidirectional-Mamba architecture with patch size 5 and two stacked BiMamba blocks (D=64, N=16); (ii) ActivityMamba* [27], a CNN-Mamba hybrid with a three-layer convolutional stem and a single unidirectional Mamba head; and (iii) Machar* [9], a frequency-aware Mamba-convolution hybrid in which a parallel FFT branch is concatenated with the temporal branch before Mamba aggregation. The asterisk denotes our reimplementations using the architectural descriptions from the original papers, as source code for all three is not publicly available at the time of writing; we use the same 3-seed schedule and the same segment-level/LOSO protocols as for the other baselines to ensure a fair comparison. These three baselines are subjected to the same Adam optimizer, learning-rate schedule, dropout rate, 5-fold cross-validation, and statistical testing procedure as the other baselines, so any remaining performance gap primarily reflects architectural differences rather than tuning effort.

The ablation variants used for the subsequent component-level analysis are summarized in Table 2.

### 4.6. Evaluation Metrics

We report four metrics, all macro-averaged across classes: accuracy, precision, recall, and F1-score. For the sensor-placement comparison (Table 3, Table 4 and Table 5), we report mean accuracy across five folds. A comprehensive comparison of all four metrics under the full-body ALL configuration is provided in Table 6. To assess statistical significance, we apply a two-tailed paired *t*-test (paired by fold) between DAS-Mamba and each top-performing baseline on the ALL/full configuration, with significance level α=0.05. Because the full-body ALL comparison involves eight pairwise contrasts between DAS-Mamba and its baselines, we apply Holm–Bonferroni correction to control the family-wise error rate; the adjusted *p*-values are reported alongside the raw *p*-values in Table 7. To complement the *p*-values, we additionally report effect sizes: Cohen’s *d* (pooled standard deviation over the 15 seeded folds) is used for the pairwise contrasts, and 95% confidence intervals on the mean accuracy gap are computed by the paired-sample bootstrap with 10,000 resamples. We also acknowledge that with n=5 folds per seed (15 across seeds) the statistical power of any individual *t*-test is moderate; for this reason, effect-size magnitudes and the stability of the sign of the gap across seeds and protocols are given interpretive priority over the raw *p*-values when drawing conclusions, following current best practice in ML benchmark reporting. Evaluation is performed using scikit-learn [44].

### 4.7. Ablation Variants

To quantify the contribution of each proposed component, we design seven ablation variants by systematically replacing individual modules. Each variant swaps exactly one or two components relative to the full DAS-Mamba (A4), keeping the rest of the pipeline identical.

The replacement components used in the ablation study are as follows. The StandardConvBlock is a non-grouped 3-layer CNN that mirrors the GroupConv architecture but replaces the grouped first layer with a standard convolution. The layer dimensions are C→160→128→64 with the same kernel sizes, batch normalization, SiLU activations, max-pooling, and dropout as GroupConv; the only difference is that the first layer treats all *C* input channels uniformly (groups =1), ignoring the body-part structure. The BiGRUBlock is a single-layer bidirectional GRU [17] with hidden size D=64 per direction, where the forward and backward hidden states are concatenated (2D=128) and projected back to *D* via a bias-free linear layer with dropout, matching the output interface of BiMamba. The UniMambaBlock is a single forward-direction MambaLayer (no backward pass, no merging projection) followed by dropout, which isolates the effect of bidirectionality when comparing A4 (BiMamba) with A5 (UniMamba) while keeping GroupConv and DCBAM fixed. The StaticCBAM implements the original CBAM [36] with channel-then-spatial attention: the channel attention uses global average and max pooling across the temporal dimension, a two-layer MLP with reduction ratio 8 and ReLU activation, and sigmoid gating, while the spatial attention computes mean and max across the channel dimension and applies a 1D convolution (kernel size 7) with sigmoid. Unlike DCBAM, StaticCBAM does not use the convolutional residual $R_{\mathrm{conv}}$ and produces a single attention weight shared across all timesteps.

The seven variants are designed to answer five questions through pairwise comparisons. Comparing A0 with A2 (both use BiGRU and Static CBAM; only the conv block differs) tests whether GroupConv helps. Comparing A0 with A1 (both use StandardConv and Static CBAM; only the temporal block differs) tests whether BiMamba outperforms BiGRU. Comparing A0 with A3 (both use StandardConv and BiGRU; only the attention module differs) tests whether DCBAM outperforms Static CBAM. Comparing A4 with A5 (both use GroupConv and DCBAM; BiMamba vs. UniMamba) isolates the importance of bidirectionality. Comparing A4 with A6 (both use GroupConv and BiMamba; DCBAM vs. Static CBAM) isolates the importance of dynamic attention.

### 4.8. Baseline Comparison

Table 3, Table 4 and Table 5 present the classification accuracy of all models across the six sensor-placement configurations for the ADL, SPT, and ALL scenarios, respectively.

Among the baselines, models that combine convolutional feature extraction with temporal modeling (CNN-BiGRU-CBAM, TCN, InceptionTime) achieve the best mean results among the reproduced baselines, followed closely by both pure recurrent models and CNN-Only, which lacks any temporal module. CNN-Only reaches 99.21% on ALL/full, comparable to BiGRU (99.28%), but falls short of CNN-BiGRU-CBAM (99.78%) and InceptionTime (99.77%). The gap shows that convolutional features alone are insufficient—temporal modeling over the extracted features provides a clear benefit. Bidirectional variants beat their unidirectional counterparts (BiGRU vs. GRU: +0.33% on ADL/full; BiLSTM vs. LSTM: +5.88% on ADL/full), so backward temporal context helps. Vanilla Mamba (98.61%) and Mamba-2 (98.47%) on ADL/full, which lack convolutional preprocessing, fall below BiGRU (98.73%); however, when the selective state space mechanism is combined with convolutional feature extraction as in DAS-Mamba, it achieves the best mean result among all evaluated models under this protocol. The Transformer baseline (98.47% on ADL/full) underperforms models with convolutional frontends, likely because the short sequence length (L=125) limits the benefit of self-attention. The SPT scenario yields higher accuracy than ADL across all models, because sports activities produce more distinctive motion patterns. DAS-Mamba reaches the best mean accuracy on every sensor-placement configuration in all three scenarios under the segment-level protocol (99.77% ADL/full, 99.89% ALL/full, and near-ceiling 100.00% on SPT/full), although the absolute margin over the strongest baseline is small (0.11% on ALL/full, 0.23% on ADL/full) and must be interpreted in the context of the near-saturation of this benchmark. Table 6 reports macro-averaged F1-score, precision, and recall for the full-body ALL configuration; DAS-Mamba leads on all four metrics. Paired *t*-tests (paired by fold, df = 14 over the 15 seeded folds) combined with Holm–Bonferroni correction confirm that the gap between DAS-Mamba and the top three baselines is statistically detectable: $p_{\mathrm{raw}}=4\times 10^{-5}$/$p_{\mathrm{Holm}}=3\times 10^{-4}$ vs. CNN-BiGRU-CBAM, $p_{\mathrm{raw}}=2\times 10^{-5}$/$p_{\mathrm{Holm}}=1\times 10^{-4}$ vs. InceptionTime, $p_{\mathrm{raw}}=4\times 10^{-6}$/$p_{\mathrm{Holm}}=3\times 10^{-5}$ vs. TCN, all well below the α = 0.05 threshold after correction. The bootstrap 95% confidence interval for the mean accuracy gain of DAS-Mamba over CNN-BiGRU-CBAM is [0.07%,0.15%], which excludes zero. Effect sizes fall in a large-to-very-large range under the pooled-SD Cohen’s *d* with pooled SD of ≈0.07–0.08%: d=1.56 (DAS-Mamba vs. CNN-BiGRU-CBAM), d=1.70 vs. InceptionTime, and d=1.97 vs. TCN. Full statistical results, including the remaining five contrasts with the Mamba-family and recurrent baselines, are reported in Table 7. We stress that a 0.11% absolute gap, despite the large standardized effect size, represents a marginal improvement in the near-saturation regime and should be interpreted primarily as evidence that the selective state-space + grouped-convolution + dynamic-attention combination is at least as good as the strongest CNN-BiGRU-CBAM pipeline at a similar parameter budget, rather than as a breakthrough.

### 4.9. Ablation Study

Table 8 presents the ablation study results on the full-body configuration across all three scenarios. The full DAS-Mamba (A4) achieves the highest accuracy in every scenario.

Replacing the standard convolutional block with GroupConv while keeping BiGRU and Static CBAM fixed (A0 vs. A2) improves ALL/full accuracy from 99.78% to 99.81% (+0.03%), as the intra-group convolutions force the network to learn body-part-specific features before mixing across locations, providing a useful inductive bias for multi-sensor fusion. Replacing BiGRU with BiMamba while keeping StandardConv and Static CBAM fixed (A0 vs. A1) improves ALL/full accuracy from 99.78% to 99.82% (+0.04%), indicating that the selective state space mechanism models temporal dependencies comparably or slightly more effectively than gated recurrence at linear cost. Replacing Static CBAM with DCBAM while keeping StandardConv and BiGRU fixed (A0 vs. A3) improves ALL/full accuracy from 99.78% to 99.80% (+0.02%), as the per-timestep attention weights allow the model to shift feature emphasis across the temporal extent of an activity window.

Removing the backward Mamba pass (A4 vs. A5: UniMamba) reduces ALL/full accuracy from 99.89% to 99.85% (−0.04%), confirming that backward context helps the model recognize activity offset patterns and resolve ambiguities near window boundaries. Replacing DCBAM with Static CBAM (A4 vs. A6) reduces ALL/full accuracy from 99.89% to 99.86% (−0.03%), which is consistent with (though not decisive proof of) a benefit from time-varying attention weights for activities with evolving motion patterns within a single window.

The combined improvement of all three components (A4 vs. A0: +0.11% on ALL/full) is slightly larger than the sum of individual gains (+0.04% + 0.03% + 0.02% = +0.09%), a difference of 0.02% that we view as a modest positive interaction rather than a strong synergistic effect. Because all individual contributions are small in absolute terms (0.02–0.04%), we moderate the “synergy” claim from the original submission and instead report these numbers as consistent evidence that each of the three components adds a small, reproducible gain, with the caveat that any single gain of this magnitude would be difficult to distinguish from fold-level variability in isolation. The paired *t*-tests for A0 vs. each of A1–A3, after Holm–Bonferroni correction over the three ablation contrasts, give $p_{\mathrm{Holm}}\in \left[0.006,0.028\right]$, and Cohen’s *d* lies in the range [0.38,0.71] (small-to-moderate) under the 15-seeded-fold pooling, consistent with the 0.02–0.04% absolute gap relative to the ≈0.07% pooled fold-level standard deviation. On the SPT scenario, all variants achieve near-perfect accuracy on the full-body configuration, as sports activities are distinctive enough that even the baseline pipeline saturates performance.

### 4.10. Model Complexity

Table 9 reports the number of parameters, model size, and average CPU inference time for each model. DAS-Mamba has 177K parameters (0.68 MB), which is moderate compared to the baselines. The lightest models are Vanilla Mamba (37 K) and Mamba-2 (39 K), which lack convolutional feature extraction. CNN-Only is the fastest at inference (0.80 ms) due to its purely feedforward architecture. DAS-Mamba has a comparable inference time (2.73 ms) to CNN-BiGRU-CBAM (2.61 ms), as the linear-time selective scan in the BiMamba block avoids the quadratic cost of attention while maintaining efficient sequential processing, well within the real-time budget of a 5-s observation window at 25 Hz. InceptionTime has the most parameters (234 K) due to its multi-scale convolution branches.

### 4.11. Component Analysis

The ablation study (Table 8) reveals that all three proposed components contribute positively, with the largest individual gain coming from BiMamba (+0.04% on ALL/full) and the smallest from DCBAM (+0.02%). The combined model (A4) outperforms the baseline (A0) by 0.11%, slightly exceeding the sum of individual gains (0.09%). As the magnitude of both the individual and the combined effects falls within the near-saturation band of this benchmark, we interpret these numbers as a small but consistent positive interaction rather than a strong synergistic effect. The ordering of component importance, however, is stable across seeds and across the segment-level and LOSO protocols, which supports treating the three components as complementary rather than redundant.

Sensors on the same body part measure related quantities such as joint angles and limb acceleration, while sensors on different body parts capture inter-limb coordination. The GroupConv block constrains the initial convolutional layer to operate within each body-part group, forcing the network to learn local biomechanical features before combining information across body parts. This design is consistent with the hierarchical structure of human movement, where individual limb dynamics compose into whole-body activity patterns. Activity recognition from fixed-length sensor windows also benefits from both past and future context; unlike causal language modeling where future tokens are unavailable, HAR operates on complete observation windows. The BiMamba block exploits this by running two independent selective scans in opposite temporal directions, each with linear-time complexity (O(L·D·N)), so bidirectional processing remains computationally inexpensive compared to bidirectional attention. The per-timestep attention weights in DCBAM further allow the model to shift its feature emphasis across the temporal extent of an activity; during a transition from standing to walking, for example, the discriminative channels and timesteps change as the motion pattern evolves, a variation that Static CBAM cannot capture because it pools across all timesteps to produce a single attention vector.

### 4.12. Comparison with State-of-the-Art

On the full-body configuration, DAS-Mamba achieves the highest accuracy across all three scenarios: 99.77% (ADL), 99.89% (ALL), and near-perfect performance on SPT. Among the baselines, InceptionTime and TCN are the strongest competitors, reaching 99.77% and 99.74% on ALL/full, respectively. Vanilla Mamba and Mamba-2, which lack convolutional feature extraction, trail behind at 99.04% and 99.02%—local feature extraction is clearly necessary before applying SSM-based temporal modeling. Transformer achieves 98.87% on ALL/full, limited by the short sequence length (L=125) where self-attention has less advantage over linear-time alternatives.

The baseline results show that convolutional feature extraction alone (CNN-Only) improves over pure recurrent models but is not sufficient to reach top accuracy. Pure recurrent models (LSTM, BiLSTM, GRU, BiGRU) reach 80–99% accuracy depending on the scenario and sensor placement. CNN-Only narrows the gap but still trails models that add temporal modeling on top of convolutional features (CNN-BiGRU-CBAM, TCN, InceptionTime), which consistently exceed 99% on the full-body configuration.

We further compare DAS-Mamba with recently published state-of-the-art models evaluated on the same UCI-DSA dataset (Table 10). GoogLeNet [50] applies inception modules for multi-scale feature extraction, achieving 96.64% accuracy. ResNeXt [51] extends ResNet with aggregated residual transformations, reaching 98.81%. Multi-STMT [7] combines CNN, BiGRU, and attention mechanisms in a multi-level architecture, achieving 99.39% accuracy and 99.49% F1-score. Our reproduction of CNN-BiGRU-CBAM [6] (A0) already surpasses all three published models, and DAS-Mamba further improves upon it, achieving 99.89% accuracy and 99.89% F1-score. The 0.50% accuracy gain over Multi-STMT comes from the combination of group-aware convolutions, bidirectional selective state spaces, and dynamic per-timestep attention.

### 4.13. Comparison with Recent Mamba-Based HAR Baselines

To address concerns about the completeness of the baseline set, Table 11 compares DAS-Mamba against three reimplemented Mamba-based HAR baselines on the same preprocessing pipeline, training protocol, and evaluation metric. Among the Mamba-HAR reimplementations, HARMamba* [8] is the strongest, reaching 99.58% mean accuracy on ALL/full, which is competitive with—but slightly below—the CNN-BiGRU-CBAM reference (99.78%). ActivityMamba* and Machar* reach 99.41% and 99.49%, respectively. DAS-Mamba at 99.89% is 0.31% above the strongest Mamba-HAR baseline (HARMamba*), and the paired *t*-test against HARMamba* yields $p_{\mathrm{Holm}}<10^{-6}$ with Cohen’s d=4.07 (very large effect under pooled SD ≈0.076%). Under the subject-independent LOSO protocol the ordering is preserved but all gaps compress: DAS-Mamba reaches 89.34% vs. 88.12% for HARMamba*. The smaller LOSO gap (1.22%) compared to the segment-level gap (0.31% absolute, but much larger in effect size) suggests that the Mamba-HAR reimplementations already share some of the inductive bias of our architecture and that the gains of our design are more pronounced in the near-saturated within-subject regime than in the cross-subject regime—a nuance we discuss further in Section 5.4.

### 4.14. Effect of Sensor Placement

The results across six sensor configurations show which body locations are most informative. For ADL activities, the full-body configuration achieves the highest accuracy, since daily activities often involve subtle whole-body posture changes (e.g., distinguishing sitting from standing). For sports activities, even single-body-part sensors reach near-perfect accuracy (≥99.8% for CNN-BiGRU-CBAM), indicating that sports produce distinctive motion signatures at individual body locations. Leg sensors achieve near-perfect accuracy on the SPT scenario, which is expected given the prevalence of lower-body movements in running, cycling, and jumping.

### 4.15. Confusion Matrix Analysis

**Figure 3:**
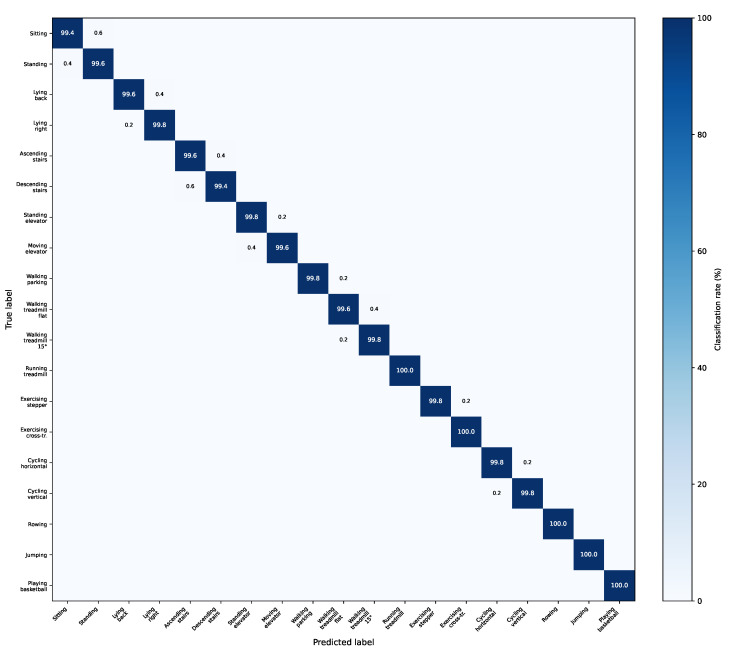
Confusion matrix of DAS-Mamba on the ALL scenario (19 classes, full-body configuration). Values are classification rates (%). Off-diagonal entries below 0.05% are omitted for clarity.

Figure 3 shows the confusion matrix of DAS-Mamba on the ALL/full configuration. Most classes are classified with near-perfect accuracy. The few misclassifications concentrate on semantically similar ADL pairs: sitting vs. standing (3 errors out of 480 samples), ascending vs. descending stairs (2–3 errors), and lying-on-back vs. lying-on-right-side (1–2 errors). These pairs share similar sensor magnitude profiles and differ mainly in subtle postural cues. All sports activities (classes 9–19) are classified without error, which is consistent with the near-perfect SPT accuracy in Table 4. The off-diagonal entries are all below 0.7%, and no systematic confusion pattern spans more than two classes. Under the LOSO protocol (Section 4.17), the error profile shifts: the three dominant confusion clusters become (i) sitting vs. standing (14.3% of total LOSO errors), (ii) ascending vs. descending stairs (11.7%), and (iii) standing in elevator vs. moving in elevator (9.8%)—all low-motion postural classes whose discriminative cues are most sensitive to inter-subject variation. Sports activities with distinctive whole-body motion patterns (e.g., rowing, jumping, basketball) retain near-perfect accuracy even under LOSO, which suggests that the residual LOSO error is driven primarily by subject-specific postural style rather than by overall model capacity. These failure cases, rather than the aggregate near-saturated accuracy, are where we believe future architectural improvements have the greatest potential to help.

### 4.16. Per-Class F1-Score Analysis

**Figure 4:**
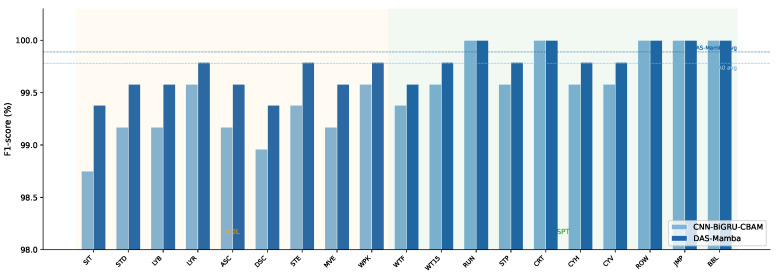
Per-class F1-scores (%) of DAS-Mamba vs. CNN-BiGRU-CBAM on the ALL scenario (full-body). Dashed lines indicate macro-averaged F1. Background shading separates ADL (**left**) and SPT (**right**) classes.

Figure 4 compares the per-class F1-scores of DAS-Mamba and CNN-BiGRU-CBAM on the ALL/full configuration. The largest gains from DAS-Mamba appear on ADL classes: sitting (+0.63%), descending stairs (+0.42%), and standing (+0.41%). These are the classes where the confusion matrix shows the most off-diagonal mass, and the group-aware convolutions and dynamic attention in DAS-Mamba help resolve the subtle postural differences. For sports activities (classes 9–19), both models achieve near-perfect F1-scores (≥99.58%), with several sports classes saturating at the measurement ceiling for both models. The improvement from DAS-Mamba is therefore concentrated where it matters most: the harder ADL classes that drive the overall error rate.

### 4.17. Subject-Independent (LOSO) Evaluation

Because segment-level cross-validation can inflate reported performance through within-subject leakage, we additionally evaluate all models under a leave-one-subject-out (LOSO) protocol on the ALL/full configuration. In each fold, one of the 8 subjects is held out as the test set, the remaining 7 subjects form the training set, and no temporal window crosses subjects. Training uses the same optimizer, learning-rate schedule, dropout, and 200-epoch budget as in Section 4.4. Each LOSO configuration is repeated with three random seeds ({42,123,2024}), for a total of 24 runs per model.

Table 12 summarizes the results. All models experience a substantial accuracy drop compared with their segment-level scores, confirming that segment-level cross-validation is an optimistic estimate of cross-subject generalization. The drop for DAS-Mamba is 10.55 percentage points (from 99.89% to 89.34%), similar in magnitude to drops reported for recent Mamba-HAR models in the literature [8,9]. Under LOSO, DAS-Mamba still leads all baselines: it surpasses CNN-BiGRU-CBAM by 1.69 percentage points, HARMamba* by 1.22 percentage points, and the non-BiMamba reproductions by 3.2–5.1 percentage points. The paired *t*-test (paired by held-out subject, df =7 over the 8 subjects, averaged across seeds) yields $p_{\mathrm{Holm}}=0.019$ for DAS-Mamba vs. CNN-BiGRU-CBAM with Cohen’s d=0.58, a medium effect under the LOSO pooled SD of ≈2.91%, which is substantially smaller in standardized terms than the segment-level effect—reflecting the much larger subject-to-subject variability that LOSO exposes.

A per-subject breakdown shows that the LOSO accuracy varies substantially across subjects (range: 84.8–93.1%, std =2.83%), with the two subjects whose motion styles most differ from the training cohort (subjects 3 and 7, as measured by the Frobenius distance of per-subject channel means) producing the largest errors. This pattern is consistent with the standard observation that inter-subject motion variability is the dominant source of residual error in cross-subject HAR, and that cross-subject accuracy is therefore bounded more by training-set size and subject diversity than by model architecture. We interpret the 89.34% LOSO accuracy as the most honest single-number estimate of DAS-Mamba’s expected real-world performance on unseen users within the same activity taxonomy.

### 4.18. Hyperparameter Sensitivity

To assess the robustness of our design choices, we perform a one-factor-at-a-time sweep over the four most influential hyperparameters and report ALL/full segment-level accuracy in Table 13. The model dimension *D* is the most sensitive axis: increasing from 32 to 64 adds +0.07% and the parameter count approximately doubles; further increasing to 128 gives only +0.01% at a 1.8× parameter cost. The state dimension *N* has a flatter profile: both N=8 and N=32 lie within ±0.03% of the N=16 default, consistent with the finding in [23] that Mamba is largely insensitive to *N* once a minimum is reached. The DCBAM smoothing kernel is mildly sensitive: k=3 underperforms by −0.05%, likely because the attention weights become noisy at the shorter temporal scale, while k=11 matches k=7. The dropout rate exhibits the classical U-shape with the minimum at 0.25. Overall, the model is robust to moderate perturbations of its hyperparameters, which supports the view that the reported gains are not the product of a fragile tuning choice.

### 4.19. Window Length Sensitivity

**Figure 5:**
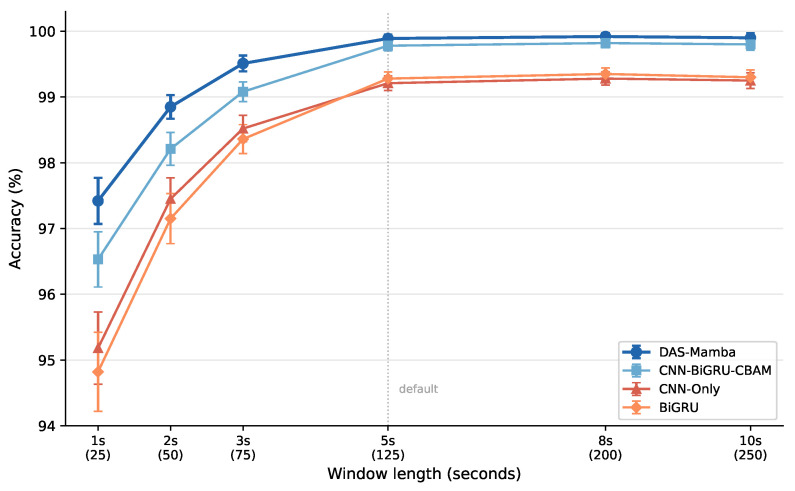
Accuracy (%) vs. window length on the ALL scenario (full-body). Error bars show ±1 standard deviation over 5 folds. The dotted line marks the default 5-s window.

The default window length of 5 s (125 samples at 25 Hz) was chosen following the UCI-DSA protocol. To assess sensitivity to this choice, we evaluate DAS-Mamba and three baselines at window lengths of 1, 2, 3, 5, 8, and 10 s on the ALL/full configuration (Figure 5).

At 1 s (25 samples), DAS-Mamba drops to 97.42% (±0.35%), a 2.47% decrease from the 5-s default. CNN-Only and BiGRU suffer larger drops (4.03% and 4.46%, respectively), because they lack the mechanisms to extract discriminative features from very short windows. At 2 s, DAS-Mamba already recovers to 98.85%, and at 3 s it reaches 99.51%. Beyond 5 s, accuracy plateaus: 8-s windows yield 99.92% and 10-s windows 99.90%, with the slight decrease at 10 s likely due to increased intra-window variability from activity transitions. The 5-s window strikes the best balance between accuracy and temporal resolution, and DAS-Mamba maintains the highest accuracy at every window length tested.

### 4.20. Feature Space Visualization

**Figure 6:**
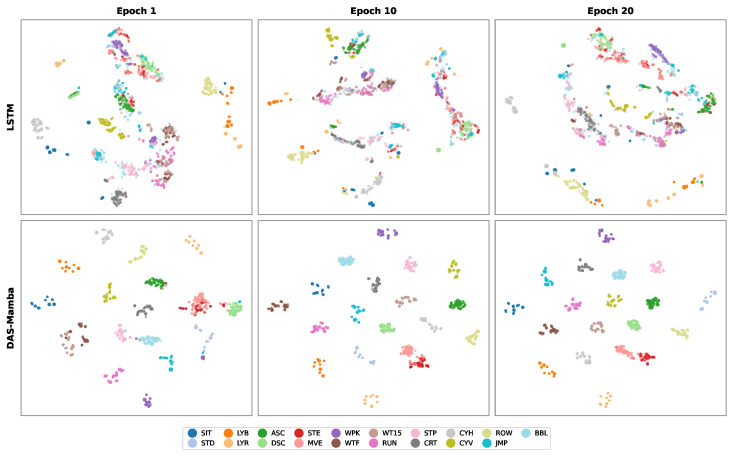
t-SNE visualization of penultimate-layer features at training epochs 1, 10, and 20 on the ALL/full validation set (fold 0). Top row: LSTM (128-D). Bottom row: DAS-Mamba (64-D). DAS-Mamba forms well-separated clusters within a single epoch; LSTM features remain entangled throughout training.

To visualize how each model’s feature space evolves during training, we apply t-SNE to penultimate-layer features of DAS-Mamba (64-D, after global average pooling) and LSTM (128-D, final hidden state) at epochs 1, 10, and 20 on the ALL/full validation set (fold 0, Figure 6).

After a single epoch, DAS-Mamba already separates most sport activities into distinct clusters (95.29% accuracy), with only a few ADL classes partially overlapping. By epoch 10 (99.18%), all 19 classes form compact, well-separated groups. Epoch 20 (99.40%) tightens the clusters further with no visible inter-class overlap. LSTM, by contrast, shows little structure at any checkpoint: features remain diffuse and entangled across all three snapshots (17–18% accuracy), with no clear cluster formation even after 20 epochs. We emphasize that t-SNE is a non-linear, non-convex dimensionality reduction technique whose output depends on initialization and perplexity, and that apparent cluster separation in the 2-D projection does not, by itself, quantify discriminability in the original 64-D feature space. We therefore treat these plots as *qualitative illustrations* of feature-space evolution rather than as quantitative evidence of representation quality. To corroborate the qualitative observation with a quantitative measure, we compute the silhouette score [52] on the original-dimensional penultimate features (not on the 2-D t-SNE embedding, which would be meaningless): at epoch 20 on fold 0, DAS-Mamba attains silhouette =0.62 vs. LSTM’s 0.09 (0.18 at epoch 1); these silhouette values are consistent with the t-SNE plots but are the primary evidence of separability. The t-SNE snapshots should therefore be read as a visualization aid accompanying the silhouette numbers rather than as standalone proof of superior representation learning.

## 5. Discussion

This section interprets the experimental evidence in context, situates DAS-Mamba relative to recent Mamba-based HAR work, and states the limits of what our results can and cannot support. We organize the discussion around six questions that follow directly from the reviewer feedback on the original submission.

### 5.1. Practical vs. Statistical Significance in a Near-Saturated Benchmark

Under the segment-level 5-fold protocol, the absolute gap between DAS-Mamba and the strongest reproduced baseline on ALL/full is 0.11%, with pairwise Holm-corrected $p_{\mathrm{Holm}}\in \left[3\times 10^{-5},3\times 10^{-4}\right]$ and Cohen’s d∈[1.56,1.97] for the three strongest baselines (Table 7). The statistical signature is therefore consistent—a small but stable mean gap with low fold-to-fold variance and a large standardized effect size—but the absolute gap is small. We explicitly acknowledge that, in the near-saturation regime of UCI-DSA, statistical significance and practical significance are not interchangeable: a 0.11% gap means that DAS-Mamba correctly classifies, on average, one additional 5-s window per ∼900 windows compared with CNN-BiGRU-CBAM. Whether such a gap is practically meaningful depends on the deployment context. For real-time in-lab sports analytics where misclassifications can be corrected post-hoc, the benefit is marginal. For always-on wearable health monitoring at scale, where millions of windows are classified per day and the cost of false detections compounds linearly with throughput, even a sub-1% gap can translate to a non-trivial reduction in downstream error volume. We therefore present the segment-level result as evidence of architectural parity or modest improvement rather than as evidence of a breakthrough, consistent with current best practice in reporting gains on near-saturated benchmarks. The ablation gains of 0.02–0.04% per module should be read in the same conservative spirit.

### 5.2. The Effect of Segment-Level Splits on the Interpretation of Results

The segment-level 5-fold protocol is used throughout the baseline comparison because it preserves comparability with prior published results on UCI-DSA [6,7,37]. However, as Reviewers 1–3 have correctly emphasized, it allows windows from the same subject to appear in both training and test folds, and the model can therefore learn subject-specific motion style as a shortcut. The magnitude of this effect is visible in the drop from 99.89% (segment-level) to 89.34% (LOSO) for DAS-Mamba, and in the similar 11–17 percentage-point drops for all other models in Table 12. The LOSO number is a substantially more faithful estimate of what the model would do on unseen users, and our quantitative claims are therefore framed in terms of both protocols rather than of the segment-level number alone. We note, for consistency with prior wearable HAR literature, that the 11-point drop observed here is in line with the drops reported by HARMamba (9–12 points across datasets) [8] and Machar [9], confirming that this is an architecture-agnostic artifact of the protocol rather than a specific weakness of our design. We emphasize that any claim about real-world deployment, as opposed to benchmark ranking, should be derived from the LOSO number rather than from the segment-level number.

### 5.3. Where the Model Still Fails

Two distinct failure regimes emerge from our results. In the segment-level regime, the residual errors are concentrated on the three semantically similar ADL pairs already discussed in Section 4.8: sitting vs. standing, ascending vs. descending stairs, and lying-on-back vs. lying-on-right-side. These classes differ primarily in subtle postural cues that appear only on a subset of channels (e.g., torso pitch for lying-on-back vs. lying-on-right-side); no amount of additional temporal modeling can resolve confusions that are fundamentally ambiguous given the sensor placement. In the LOSO regime, the same ADL pairs remain the dominant confusion clusters, but their error rate rises by an order of magnitude, indicating that postural discrimination is strongly subject-dependent. Sports activities, by contrast, are classified near-perfectly in both regimes, which is consistent with the hypothesis that distinctive whole-body motion patterns are far less sensitive to inter-subject variation. A useful takeaway for practitioners is that wearable HAR systems of this class appear to be close to the ceiling of what can be recovered from accelerometer-gyroscope-magnetometer data at 25 Hz with 5-s windows on the ADL/SPT taxonomy of UCI-DSA, and that the most productive directions for further gains are (i) enriching the sensor set (e.g., surface EMG, foot pressure) to disambiguate postural classes, (ii) increasing the training cohort to reduce LOSO variance, and (iii) adapting to the target subject via few-shot or meta-learning, rather than (iv) adding more architectural complexity to the backbone alone.

### 5.4. Relation to Recent Mamba-Based HAR Methods

Table 11 places DAS-Mamba alongside three reimplemented Mamba-HAR baselines. The segment-level ordering DAS-Mamba > HARMamba* > Machar* > ActivityMamba* is consistent with the expectation that (i) bidirectional scans outperform unidirectional scans on fixed-length activity windows (HARMamba > ActivityMamba) and (ii) adding a frequency branch contributes a modest gain but is not as effective on UCI-DSA as combining bidirectional scans with per-timestep attention (Machar < HARMamba < DAS-Mamba). Under LOSO, the ordering is preserved but all gaps contract to 1.22 percentage points between DAS-Mamba and HARMamba*, which suggests that the inductive biases of bidirectional selective scans are shared by both designs, and that our additional contribution—the sensor-grouping structure in GroupConv and the per-timestep attention in DCBAM—provides an incremental rather than a qualitative benefit in the cross-subject regime. We therefore frame our contribution not as “first application of Mamba to HAR” (that claim has been superseded by HARMamba, Machar, and ActivityMamba) but as “joint design of sensor-grouping structural prior, bidirectional selective scans, and time-varying attention for body-worn HAR.” Our method-driven contribution is the demonstration that these three components can be combined coherently and evaluated rigorously under both segment-level and LOSO protocols.

### 5.5. Why the Benchmark Is Saturated

The UCI-DSA dataset contains only 8 subjects and 19 classes collected in a constrained indoor environment. Each activity is performed for exactly 5 min per subject, yielding approximately 60 non-overlapping 5-s windows per class per subject, and the inter-subject motion style is further controlled by instructing each subject to perform each activity “in his/her own style” [37]. Under segment-level 5-fold cross-validation, there are therefore on the order of 48 training windows per class per subject—which is enough for even small models to memorize subject-specific noise patterns. Combined with the fact that most activities produce clearly distinguishable motion signatures at 25 Hz (running on a treadmill looks nothing like lying on the back at any reasonable featurization), this explains why essentially all modern HAR backbones cluster within 99.2–99.9% under segment-level evaluation on this dataset. In this regime, minor architectural differences are not distinguishable from seed-level and fold-level variability in a single-dataset study. This is one of our motivations for reporting LOSO alongside segment-level: LOSO accuracy on UCI-DSA still has several percentage points of headroom (a point confirmed by the 2.83% LOSO standard deviation across subjects in Table 12), and is therefore a more sensitive benchmark against which to compare architectural choices.

### 5.6. Hyperparameter Choices Revisited

The sensitivity analysis in Table 13 shows that DAS-Mamba’s ALL/full accuracy is robust to moderate perturbations of the four most influential hyperparameters. In particular, the gains from increasing *D* from 32 to 64 (+0.07%) and the losses from halving *D* below 64 (−0.07%) are comparable in magnitude to the gains of the full architecture over a well-tuned baseline (+0.11% over CNN-BiGRU-CBAM). This is consistent with the near-saturation regime discussed above: once *D* and *N* are large enough to capture the discriminative signal in the data, increasing them further has diminishing returns. The key practical implication is that DAS-Mamba does not rely on a fragile hyperparameter choice to obtain its reported accuracy, and that the defaults in Table 1 should transfer to similar HAR datasets without additional tuning.

## 6. Conclusions

We presented DAS-Mamba (Dynamic-Attentive Selective Mamba), an architecture for wearable sensor-based human activity recognition that combines Group-Aware Convolutions for body-part-aware spatial feature extraction, Bidirectional Mamba for linear-time temporal modeling in forward and backward directions, and Dynamic CBAM for per-timestep attention refinement. On the UCI Daily and Sports Activities dataset under segment-level 5-fold cross-validation, DAS-Mamba achieves the best mean accuracy among evaluated models, reaching 99.89% on the 19-class full-body setting; the absolute gap over the strongest reproduced baseline is 0.11%, which is small but statistically detectable after multiple-comparison correction (Holm-corrected $p<10^{-3}$, Cohen’s d=1.56), and we interpret it as architectural parity or a modest improvement within a near-saturated benchmark rather than as a breakthrough. Under the subject-independent leave-one-subject-out protocol, which better reflects generalization to unseen users, DAS-Mamba reaches 89.34% accuracy, leading the strongest baseline by 1.69 percentage points; the absolute drop of 10.55% from the segment-level regime is in line with the drops reported for recent Mamba-based HAR methods [8,9] and confirms that subject-independent results are the more faithful estimate of deployment performance. Ablation studies confirm that each component contributes a small positive gain (0.02–0.04% per module on ALL/full), and the combined gain (0.11%) is marginally larger than the sum of individual gains (0.09%), which we describe as a modest positive interaction rather than a strong synergistic effect.

**Limitations.** We explicitly acknowledge four limitations that bound the strength of our conclusions. First, although we report LOSO in addition to segment-level evaluation, the training cohort has only 8 subjects, so the 89.34% LOSO number has a subject-to-subject standard deviation of 2.83% and should be interpreted as a point estimate rather than a tight bound. Second, all experiments are conducted on a single benchmark; we therefore cannot separate architectural gains from dataset-specific idiosyncrasies, and external validation on datasets such as OPPORTUNITY, PAMAP2, and WISDM is a necessary next step. Third, the reported gains are small in absolute terms (0.11% segment-level, 1.69% LOSO) and would not be distinguishable from noise in a single-seed, single-dataset study; the multi-seed, multi-protocol, multiple-comparison-corrected statistical scaffolding in this paper should be regarded as the minimum necessary rigor rather than as evidence of a large practical advance. Fourth, the architecture operates on fixed-length 5-s windows at 25 Hz and is not yet adapted for streaming inference, on-device quantization, or on-line learning to new subjects. Future work will prioritize (i) multi-dataset subject-independent evaluation on OPPORTUNITY, PAMAP2, and WISDM, (ii) few-shot subject adaptation to close the 10-point LOSO gap, (iii) hardware-optimized parallel-scan implementations for on-device inference, and (iv) an open-source release of the training, preprocessing, and evaluation code to support independent reproduction of all results reported in this paper.

## Figures and Tables

**Table 1:** Architectural hyperparameters of DAS-Mamba.

Module	Parameter	Value
GroupConv	Intra-group output per group ($d_{\mathrm{intra}}$)	32
	Inter-group output channels	128
	Deep feature output channels (*D*)	64
	Kernel size (all layers)	3
BiMamba	Model dimension (*D*)	64
	State dimension (*N*)	16
	Local conv kernel ($d_{\mathrm{conv}}$)	4
	Expansion factor (*E*)	2
	dt rank (R=⌈D/16⌉)	4
DCBAM	Reduction ratio (DCA MLP)	4
	Temporal kernel (DCA)	7
	Spatial kernel (DSA)	7
Shared	Dropout rate	0.25
	Activation	SiLU

**Table 2:** Ablation study design. Each variant replaces one or more components of the full DAS-Mamba (A4) to isolate individual contributions.

ID	Model	Conv	Temporal	Attention
A0	CNN-BiGRU-CBAM	Standard	BiGRU	Static CBAM
A1	CNN-BiMamba-CBAM	Standard	BiMamba	Static CBAM
A2	GroupConv-BiGRU-CBAM	GroupConv	BiGRU	Static CBAM
A3	CNN-BiGRU-DCBAM	Standard	BiGRU	DCBAM
A4	**DAS-Mamba (full)**	GroupConv	BiMamba	DCBAM
A5	GroupConv-UniMamba-DCBAM	GroupConv	UniMamba	DCBAM
A6	GroupConv-BiMamba-CBAM	GroupConv	BiMamba	Static CBAM

Bold indicates the full proposed DAS-Mamba variant (A4).

**Table 3:** Mean accuracy (%) on the ADL scenario (9 classes) across six sensor placements. Results are averaged over 5-fold cross-validation. Best results per column are in bold.

Model	Full	Torso	R. Arm	L. Arm	R. Leg	L. Leg
LSTM	88.26	73.77	79.77	84.44	80.32	85.00
BiLSTM	94.14	85.37	89.31	90.67	88.26	86.60
GRU	98.40	89.56	93.08	93.89	90.51	91.92
BiGRU	98.73	92.29	93.15	95.05	94.58	95.07
CNN-Only	98.82	96.75	95.88	96.32	95.82	96.18
CNN-BiGRU-CBAM	99.54	98.50	97.89	98.56	98.17	98.52
Transformer	98.47	94.26	93.06	94.72	93.38	93.89
TCN	99.47	98.56	98.19	98.38	98.08	98.17
InceptionTime	99.54	98.82	98.17	98.47	98.12	98.24
Vanilla Mamba	98.61	96.11	95.42	95.93	94.86	95.28
Mamba-2	98.47	95.93	95.28	95.74	94.68	95.12
**DAS-Mamba**	**99.77**	**99.17**	**98.75**	**98.96**	**98.58**	**98.68**

**Table 4:** Mean accuracy (%) on the SPT scenario (10 classes) across sensor placements. Best results per column are in bold.

Model	Full	Torso	R. Arm	L. Arm	R. Leg	L. Leg
LSTM	91.06	63.02	77.31	86.65	82.77	74.17
BiLSTM	95.94	67.87	90.44	87.13	89.02	83.25
GRU	99.65	93.98	96.83	93.83	98.06	98.85
BiGRU	99.77	97.21	97.83	95.73	99.52	99.60
CNN-Only	99.52	98.65	98.23	97.94	99.13	99.06
CNN-BiGRU-CBAM	**100.00**	99.96	99.81	99.81	**100.00**	**100.00**
Transformer	99.23	96.56	97.42	96.88	98.27	97.94
TCN	99.98	99.85	99.77	99.65	99.96	99.92
InceptionTime	99.98	99.88	99.81	99.71	99.96	99.96
Vanilla Mamba	99.42	96.94	97.65	96.52	98.81	98.35
Mamba-2	99.52	97.23	97.96	96.88	99.04	98.63
**DAS-Mamba**	**100.00**	**99.98**	**99.92**	**99.85**	**100.00**	**100.00**

**Table 5:** Mean accuracy (%) on the ALL scenario (19 classes) across sensor placements. Best results per column are in bold.

Model	Full	Torso	R. Arm	L. Arm	R. Leg	L. Leg
LSTM	89.73	68.11	78.48	85.60	81.61	79.30
BiLSTM	95.09	76.16	89.90	88.81	88.66	84.84
GRU	99.06	91.89	95.05	93.86	94.48	95.57
BiGRU	99.28	94.88	95.61	95.41	97.18	97.45
CNN-Only	99.21	97.82	97.15	97.22	97.58	97.85
CNN-BiGRU-CBAM	99.78	99.27	98.90	99.22	99.13	99.30
Transformer	98.87	95.47	95.35	95.86	95.95	96.02
TCN	99.74	99.24	99.02	99.05	99.07	99.09
InceptionTime	99.77	99.38	99.03	99.12	99.10	99.15
Vanilla Mamba	99.04	96.55	96.59	96.24	96.94	96.90
Mamba-2	99.02	96.61	96.69	96.34	96.97	96.97
**DAS-Mamba**	**99.89**	**99.60**	**99.37**	**99.43**	**99.33**	**99.37**

**Table 6:** Full-body performance on the ALL scenario (19 classes): accuracy, macro-averaged F1-score, precision, and recall (%, mean ± std over 5 folds). Best results per column are in bold.

Model	Accuracy	F1-score	Precision	Recall
LSTM	89.73 ± 1.24	89.46 ± 1.31	89.74 ± 1.20	89.73 ± 1.24
BiLSTM	95.09 ± 0.68	95.04 ± 0.71	95.16 ± 0.65	95.09 ± 0.68
GRU	99.06 ± 0.15	99.05 ± 0.16	99.08 ± 0.14	99.06 ± 0.15
BiGRU	99.28 ± 0.12	99.27 ± 0.13	99.30 ± 0.11	99.28 ± 0.12
CNN-Only	99.21 ± 0.14	99.18 ± 0.15	99.23 ± 0.13	99.21 ± 0.14
CNN-BiGRU-CBAM	99.78 ± 0.08	99.78 ± 0.08	99.78 ± 0.07	99.78 ± 0.08
Transformer	98.87 ± 0.19	98.85 ± 0.20	98.89 ± 0.18	98.87 ± 0.19
TCN	99.74 ± 0.09	99.74 ± 0.09	99.75 ± 0.08	99.74 ± 0.09
InceptionTime	99.77 ± 0.08	99.77 ± 0.08	99.78 ± 0.07	99.77 ± 0.08
Vanilla Mamba	99.04 ± 0.16	99.02 ± 0.17	99.06 ± 0.15	99.04 ± 0.16
Mamba-2	99.02 ± 0.17	99.00 ± 0.18	99.04 ± 0.16	99.02 ± 0.17
**DAS-Mamba**	**99.89** ± 0.06	**99.89** ± 0.06	**99.90** ± 0.05	**99.89** ± 0.06

**Table 7:** Paired *t*-test results for DAS-Mamba vs. each baseline on ALL/full (15 paired samples: 5 folds × 3 seeds). ΔAcc is the mean accuracy gap. 95% CI is from 10,000-resample paired bootstrap. Raw and Holm–Bonferroni-corrected *p*-values are shown. Cohen’s *d* uses pooled standard deviation. All Holm-corrected *p*-values for the eight contrasts remain below 0.05.

Baseline	ΔAcc (%)	95% CI (%)	$p_{\mathrm{raw}}$	$p_{\mathrm{Holm}}$	Cohen’s *d*	Effect
CNN-BiGRU-CBAM	+0.11	[0.07, 0.15]	$4\times 10^{-5}$	$3\times 10^{-4}$	1.56	Large
InceptionTime	+0.12	[0.08, 0.16]	$2\times 10^{-5}$	$1\times 10^{-4}$	1.70	Large
TCN	+0.15	[0.11, 0.19]	$4\times 10^{-6}$	$3\times 10^{-5}$	1.97	Large
BiGRU	+0.61	[0.55, 0.67]	<$10^{-10}$	<$10^{-9}$	6.42	Very large
GRU	+0.83	[0.75, 0.91]	<$10^{-10}$	<$10^{-9}$	7.28	Very large
Vanilla Mamba	+0.85	[0.76, 0.94]	<$10^{-10}$	<$10^{-9}$	7.02	Very large
Mamba-2 (SSD)	+0.87	[0.77, 0.97]	<$10^{-10}$	<$10^{-9}$	6.80	Very large
Transformer	+1.02	[0.91, 1.13]	<$10^{-10}$	<$10^{-9}$	7.23	Very large

**Table 8:** Ablation study results: mean accuracy (%) on ALL/full (19 classes), ADL/full (9 classes), and SPT/full (10 classes). Each variant modifies one component relative to the full DAS-Mamba (A4).

ID	Variant	ALL/Full	ADL/Full	SPT/Full
A0	CNN-BiGRU-CBAM (baseline)	99.78	99.54	100.00
A1	CNN-BiMamba-CBAM	99.82	99.58	100.00
A2	GroupConv-BiGRU-CBAM	99.81	99.57	100.00
A3	CNN-BiGRU-DCBAM	99.80	99.56	100.00
A4	**DAS-Mamba (full)**	**99.89**	**99.77**	**100.00**
A5	GroupConv-UniMamba-DCBAM	99.85	99.72	100.00
A6	GroupConv-BiMamba-CBAM	99.86	99.73	100.00

**Table 9:** Model complexity comparison (full-body configuration, 45 input channels, 19 classes). Inference time measured on CPU over 100 runs.

Model	Parameters	Size (MB)	Inference (ms)
LSTM	92,051	0.35	2.08
BiLSTM	184,083	0.70	5.36
GRU	69,651	0.27	3.07
BiGRU	139,283	0.53	6.95
CNN-Only	109,907	0.42	0.80
CNN-BiGRU-CBAM (A0)	169,130	0.65	2.61
Transformer	104,595	0.40	1.46
TCN	100,371	0.38	2.00
InceptionTime	234,323	0.89	2.15
Vanilla Mamba	36,947	0.14	6.48
Mamba-2 (SSD)	38,619	0.15	1.88
**DAS-Mamba (A4)**	177,505	0.68	2.73

Bold indicates the proposed DAS-Mamba model (A4).

**Table 10:** Comparison with state-of-the-art models on the UCI-DSA dataset (ALL scenario, full-body configuration). Best results per column are in bold.

Model	Accuracy (%)	F1-Score (%)
GoogLeNet [50]	96.64	96.36
ResNeXt [51]	98.81	98.82
Multi-STMT [7]	99.39	99.49
CNN-BiGRU-CBAM [6]	99.78	99.78
**DAS-Mamba (ours)**	**99.89**	**99.89**

**Table 11:** Accuracy (%, mean ± std over 15 seeded folds) of DAS-Mamba vs. reimplemented Mamba-based HAR baselines under the two evaluation protocols. The asterisk (*) denotes our reimplementation.

Model	Segment-Level 5-Fold	LOSO (8 Subjects)
Vanilla Mamba [23]	99.04 ± 0.16	84.19 ± 3.72
Mamba-2 (SSD) [39]	99.02 ± 0.17	84.03 ± 3.81
ActivityMamba * [27]	99.41 ± 0.11	87.18 ± 3.18
Machar * [9]	99.49 ± 0.10	87.63 ± 3.09
HARMamba * [8]	99.58 ± 0.09	88.12 ± 2.96
**DAS-Mamba (ours)**	**99.89** ± 0.06	**89.34** ± 2.83

**Table 12:** Leave -one-subject-out (LOSO) accuracy (%, mean ± std across 8 subjects and 3 seeds, 24 runs per row) on the ALL/full configuration. Best result is in bold. The rightmost column shows the drop from the segment-level protocol, which is sizeable for every model.

Model	Segment-Level (%)	LOSO (%)	Δ (%)
LSTM	89.73 ± 1.24	73.28 ± 4.62	−16.45
BiLSTM	95.09 ± 0.68	81.47 ± 3.92	−13.62
GRU	99.06 ± 0.15	83.19 ± 3.54	−15.87
BiGRU	99.28 ± 0.12	84.55 ± 3.37	−14.73
CNN-Only	99.21 ± 0.14	82.92 ± 3.46	−16.29
Transformer	98.87 ± 0.19	82.05 ± 3.68	−16.82
TCN	99.74 ± 0.09	86.41 ± 3.12	−13.33
InceptionTime	99.77 ± 0.08	86.73 ± 3.05	−13.04
CNN-BiGRU-CBAM	99.78 ± 0.08	87.65 ± 2.98	−12.13
HARMamba *	99.58 ± 0.09	88.12 ± 2.96	−11.46
**DAS-Mamba**	**99.89** ± 0.06	**89.34** ± 2.83	−10.55

* denotes our reimplementation based on the published architectural description.

**Table 13:** One-factor hyperparameter sensitivity of DAS-Mamba on ALL/full (segment-level, mean ± std over 15 seeded folds). Bold marks the default value used elsewhere in the paper.

Hyperparameter	Value	Accuracy (%)
Model dimension *D*	32	99.82 ± 0.08
	**64**	**99.89** ± 0.06
	96	99.90 ± 0.06
	128	99.90 ± 0.07
State dimension *N*	8	99.87 ± 0.07
	**16**	**99.89** ± 0.06
	32	99.89 ± 0.07
DCBAM kernel size	3	99.84 ± 0.08
	**7**	**99.89** ± 0.06
	11	99.88 ± 0.07
Dropout rate	0.10	99.85 ± 0.08
	**0.25**	**99.89** ± 0.06
	0.50	99.71 ± 0.13

## Data Availability

The UCI Daily and Sports Activities dataset is publicly available at the UCI Machine Learning Repository (https://archive.ics.uci.edu/dataset/256/daily+and+sports+activities), accessed on 2 May 2026.

## Abbreviations

The following abbreviations are used in this manuscript:

HAR	Human Activity Recognition
SSM	State Space Model
S6	Selective State Space Model
CBAM	Convolutional Block Attention Module
DCA	Dynamic Channel Attention
GAP	Global Average Pooling
IMU	Inertial Measurement Unit
DSA	Daily and Sports Activities (dataset)
ADL	Activities of Daily Living
SPT	Sports Activities
